# Can meditation improve attention in older adults? Study protocol for a 4-week proof-of-concept intervention

**DOI:** 10.1186/s40814-019-0413-x

**Published:** 2019-02-11

**Authors:** Lindsay S. Nagamatsu, Sabrina D. Ford

**Affiliations:** 1 School of Kinesiology, Western University, 3M Centre, Room 2225, London, ON N6A 5B9 Canada; 2 Department of Neuroscience, Western University, Robarts Research Institute, RRI 3203, London, ON N6A 3K7 Canada

**Keywords:** Meditation, Falls risk, Mobility, Attention, Intervention, EEG

## Abstract

**Background:**

Falls are a major health care concern for our aging population. Previous research has identified impaired sustained attention as a risk factor for falls. Recently, meditation has been shown to improve different types of attention in various populations. However, there are no studies to date examining whether meditation training can improve sustained attention and mobility in older adults.

**Methods:**

We are conducting a 4-week proof-of-concept meditation intervention. We will recruit community-dwelling older adults. Participants will be randomized into one of two groups: (1) meditation training or (2) music listening (control). All participants will complete three 20-min group sessions per week and will be encouraged to continue their practice independently on the remaining days each week. Our primary outcome measure is behavioral performance on the Sustained Attention to Response Task (SART). Our secondary and tertiary outcomes include electroencephalograms (EEG) to assess attention and cognitive processing, mobility, and executive function.

**Discussion:**

Our proof-of-concept intervention aims to examine whether meditation training can improve sustained attention in older adults, who are known to be susceptible to falls. Importantly, our research has the potential to inform future clinical trials aimed at improving mobility and reducing falls risk in our aging population.

**Trial registration:**

Clinicaltrials.gov ID NCT03417635.

## Background

Approximately 30% of community-dwelling older adults experience one or more falls per year, resulting in injuries, loss of independence, and reduced quality of life. While there are known physiological risk factors for falls, including poor balance and altered gait patterns, it is now recognized that impaired cognitive functioning is also a risk factor for falls [1, 2].

Within the broad construct of cognition, one specific domain that has been focused on in the falls literature is attention [3, 4]. Anecdotally, older adults often cite the reason for their fall as “I wasn’t paying attention.” Empirically, the relationship between attention and falls has been tested using various measures of attention, including those examining divided [5, 6], visual-spatial [7, 8], and sustained attention [9]. Particularly relevant for our current study, we previously demonstrated that the number of falls experienced over a 12-month period was negatively associated with sustained attention based on reduced performance on the Sustained Attention to Response Task (SART) [9]. The SART requires participants to respond to frequently presented targets while withholding their response to infrequent targets. Because responses are frequent and repetitive, it is easy to make errors if you are unable to continuously maintain your attention to the task. Our previous findings corroborate research implicating sustained attention in postural control; indeed, completing a sustained attention task has been shown to impair subsequent performance on a postural control task [10].

Given the importance of sustained attention for postural control, improving attention in those at-risk for falls may improve their mobility and hence reduce their falls risk. One strategy that has been previously shown to improve attention is meditation. Meditation refers to a mental practice with the goal of strengthening one’s ability to focus attention for extended periods of time [11]. There are many cited physiological, cognitive, and psychological benefits of meditation, including regulation of the stress response in humans [12], changes in brain activity [13, 14], and reductions in depressive and anxiety symptoms [15]. Importantly, there is evidence that meditation can improve attention and executive functioning in healthy young adults [14, 16, 17]. For example, one study found that participants who completed a daily meditation practice showed improved attention in just 4 days [18]. More recently, researchers have replicated these findings in healthy older adults, who significantly improved their performance on an emotional-counting Stroop task measuring attention [19]. There have also been studies that have suggested that a consistent meditation practice can act as a neuroprotective agent against cognitive decline that is typical with aging [20].

There is also emerging evidence that meditation can change electrical activity in the brain, as measured using electroencephalography (EEG). In a systematic review [21], it was found that studies looking at EEG and meditation showed an increase in alpha activity during meditation, as well as increases in P300 event-related potential (ERP) component amplitude during various attention tasks, including a Go/No go task similar to the SART. Both alpha activity and P300 amplitude are modulated by attention. Collectively, these findings have been interpreted as showing that meditation can effectively change the electrical activity in the brain to increase attentional resources.

Our proof-of-concept study aims to investigate the impact of meditation training on measures of attention and corresponding electrical signals in the brain recorded using electroencephalograms (EEG) in older adults. More specifically, we are measuring continuous EEG during resting-state (i.e., the absence of an external task) which allows us to examine alpha activity. In addition, we are measuring ERPs during the SART. ERPs can be defined as time-locked recordings of brain activity corresponding to the presentation of stimuli. This allows us to examine changes in attention processing independent of motor responses to stimuli. In our 4-week intervention, participants will be randomly assigned to either a meditation group or a music (control) group. Our mediation training will be centered on “focused-attention” (FA) meditation, where participants choose an object or sensation to focus on and monitor for any thoughts or intrusions that distract that focus [22]. Our current study focuses on FA meditation strategies, as it is recommended for beginner-level meditators [23]. FA meditation is recommended for beginners because training one’s mind to be non-judgmental and still for an extended period of time is difficult. Therefore, training to first be able to focus on one thing for an increasing period of time is recommended. Although FA meditation is the first step in a meditation practice, it has been found to increase levels of executive functioning and attention [22, 24].

We hypothesize that 4 weeks of meditation training in older adults will (1) improve attention, as measured by behavioral performance (reaction time and accuracy) on the SART, (2) lead to changes in sensory and cognitive processing of attention-demanding stimuli, as indicated by increases in amplitude and decreases in latency of both the P1 and P300 ERP components, and (3) increase levels of resting state attention, as measured by an increase in individual alpha peak frequency (iAPF) during rest from pre- to post-intervention in only the meditation group. Our work has the potential to increase our knowledge about the relationship between attention, mobility, and electrical brain activity in older adults. Further, our research may be used to inform future studies aimed at improving mobility and reducing falls risk in our aging population.

## Methods

We are conducting a proof-of-concept, 4-week meditation intervention. Our study has received ethics approval from the Health Sciences Research Ethics Board at Western University and our trial is registered at clinicaltrials.gov (ID: NCT03417635), January 30, 2018. The SPIRIT checklist has been completed.

### Participants

We will recruit older adults who meet the following inclusion criteria: (1) living independently in London, Ontario, Canada, (2) completed high school, (3) comfortable writing and reading English, (4) able to walk independently, (5) right-handed (for EEG analysis—left-handed individuals have less lateralized brain structure/function), (6) score > 6/8 on the instrumental activities of daily living scale, and (7) score > 24/30 on the Mini-Mental Status Examination. Participants will be excluded if they (1) have a diagnosis of neurodegenerative disease, (2) have a diagnosis of cognitive impairment (e.g., MCI), (3) have a diagnosis of a psychiatric condition, (4) have had a concussion in the last 12 months, (5) have had a stroke, (6) have musculoskeletal or joint disease, (7) experience dizziness or loss of balance, (8) have visual, auditory, or somatosensory impairment, or (9) have a recent history (past 2 years) of meditation practice or include a meditation component in their religious practice.

Based on our power calculation in g*power, with two groups and setting alpha = 0.05 and assuming a medium effect size [18], we would require a total of 52 participants in our study to obtain a power of 0.8075. Due to 10–15% estimated drop-out, we are aiming to recruit a total of 60 participants or 30 per group.

### Procedure

**Fig. 1:**
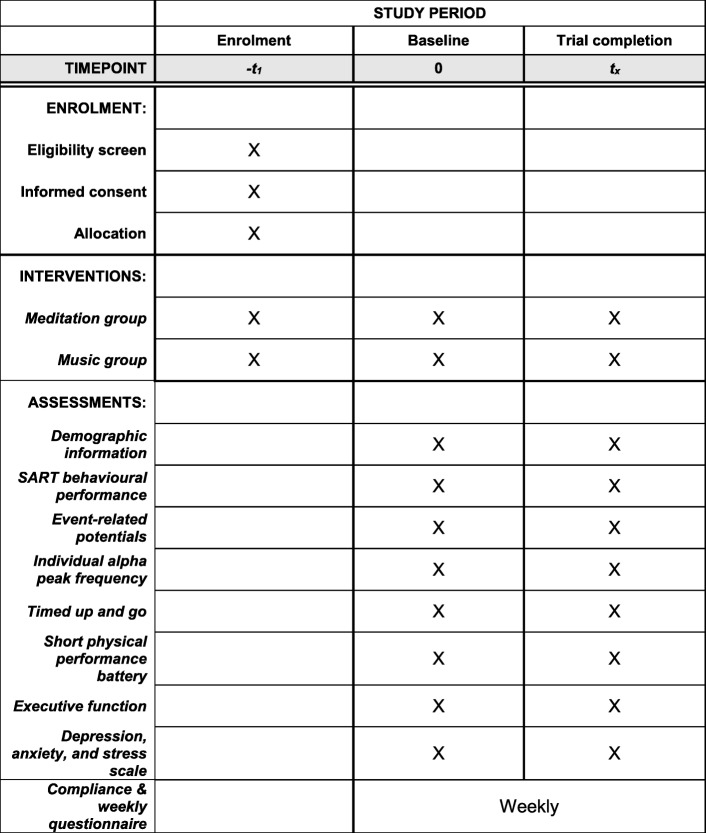
Schedule of enrolment, interventions, and assessments

See Fig. 1 for an overview of our schedule for assessments. We will advertise our study via flyers and posters in the community. Interested participants will be asked to contact us via telephone or email, where we will explain the study in more detail, answer any questions, and screen them for eligibility. If they are eligible and interested in participating, we will schedule an assessment session to obtain informed consent and conduct baseline assessments for demographic, cognitive, and physical measures. They will then be invited to complete a second session for the baseline EEG session. After all baseline measures are obtained, participants will be randomized using a random sequence generator (randomization.com) into one of two groups: meditation training or control. Randomization will be completed on an individual basis regardless of their retirement or community location. Upon completion of the 4-week intervention, all participants will complete the same assessments as baseline (demographic/cognitive/physical measures). All assessments will be completed by a blinded assessor. Baseline testing will occur within 1 month before the first session of the intervention, and the end-point testing will occur within a maximum of 7 days after the last intervention session. Participants will be allowed to discontinue the intervention at any point.

### Intervention

Participants in both groups will complete three 20-min sessions per week in a group setting (6–10 participants) with an experienced meditation leader. The meditations are being led by the graduate student who is carrying out the research. She has had a consistent meditation practice on her own for 3 years and has practiced daily for 1 year. Additionally, she has been teaching weekly meditation sessions for several months. She has experience with various types of meditation, but primarily practices the focused attention meditation type that is being used in this protocol. In addition, they will be encouraged to practice their assigned task (meditation or music listening) during the other 4 days of the week independently. The intervention will last four consecutive weeks (12 in-person sessions total). We will record attendance during each session to measure adherence.

#### Meditation training

Participants will sit in a comfortable position on a chair or on the floor. They will be guided through a focused attention practice that will focus primarily on the sensation of breathing through the nose. As the sessions progress, the practice will become less instructed and more independent with fewer reminders of the task at hand.

#### Music (control)

Participants will sit comfortably in a chair. They will be instructed to close their eyes and relax while listening to an acoustic music track. There will be no further instructions provided to participants in the control group. Music listening was chosen as our control group as it requires the same time commitment and environment as the meditation training but without specifically guiding attention. To date, there is no evidence-based or consistently used control group for meditation research [25]; therefore, we chose a calming music listening group to replicate the possible confounding variables such as socialization, taking the same time out of the day to relax, and others that are expected in the meditation group.

### Descriptive measures

Demographic information (age, sex, and education) will be ascertained via questionnaire. Cognitive status will be assessed using the Mini-Mental Status Examination (MMSE) [26]. Depression will be screened for using the Geriatric Depression Scale (GDS) [27]. Number of comorbidities will be recorded using the Functional Comorbidity Index (FCI) [28]. Instrumental activities of daily living (IADLs) will be assessed using the Lawton and Brody IADL scale [29].

### Primary outcome measure

Our primary outcome measure is behavioral performance on the sustained attention to response task (SART). Participants will view a series of equiprobable but randomly presented digits (0–9) on a computer screen and be required to press a button as quickly and accurately as possible each time a digit is presented, except they will be required to withhold their response for the number “3.” There are 14 blocks, each with 60 trials, which results in a total of 840 trials. Past experiments have used this task in older adults and have shown that this model provides a sufficient number of targets to yield significant between-group differences [9]. Response time and accuracy will be recorded. We will focus on ERPs collected from target trials.

### Secondary outcomes

During performance of the SART task, we will also record electroencephalograms (EEGs) using our Brain Vision 64 channel ActiCHamp. The EEG signal will be recorded using Brain Vision PyCorder (https://www.brainproducts.com/productdetails.php?id=43). EEG activity will be recorded relative to a scalp electrode located over the anterior frontal cortex (AFz) and will be filtered between 0.01 and 100 Hz and digitized at a rate of 500 Hz. We will also record vertical and horizontal electrooculograms to monitor eye movements. ERPs will be analyzed using ERPLAB within Matlab (https://erpinfo.org). Data will be filtered (between 0.1 and 30 Hz), segmented into epochs (− 200 to 800 msec), and cleaned from artifacts (i.e., blinks, movements). Epoched data will then be averaged by event type (target vs. non-target) and then grand averages will be created for each group and time point separately. Our main ERP measures will be mean amplitude and peak latency of the P1 and P300 components, which index sensory and cognitive processing, respectively. These values will be extracted on an individual subject basis and imported into SPSS for further statistical analysis.

We will also use EEG to record 5 min of resting-state (i.e., task-free) electrical signals. For the resting state, participants will sit comfortably with their eyes closed for 5 min. They will be instructed to remain relaxed but awake and to sit as still as possible. We will extract the alpha peak frequency (the maximum power value in the frequency spectrum between 7.5 and 12.5 Hz) for each individual. Resting-state EEG will be analyzed using Fieldtrip within Matlab (http://www.fieldtriptoolbox.org).

### Tertiary outcome measures

#### Mobility measures

We will assess general mobility using the Timed Up and Go (TUG) test [30] which requires participants to stand up from a chair, walk 3 m, return to their chair, and sit back down. The average time to complete two trials is recorded. Balance and mobility will be assessed using the Short Physical Performance Battery (SPPB) [31] which requires participants to perform a series of balance tasks, chair stands, and a 4-m walk, each of which is scored out of 4 for a maximum score of 12.

#### Executive function measures

We will assess three separate domains of executive function using pen and paper versions of each test. Selective attention and response inhibition will be measured using the Stroop color-word test. Set-shifting will be measured using the Trail Making Test (Part A and B). Working memory will be tested using the digit span test (forward and backward).

#### Other measures

Finally, once per week, participants will complete a “Weekly Questionnaire” that asks how relaxed, focused, and stressed participants feel and to measure compliance for their independent sessions. Participants will also have the opportunity to give feedback on their experience of the meditation sessions in this questionnaire. We will also measure depression, anxiety, and stress using the 21-item Depression, Anxiety, and Stress Scale [32].

### Statistical analysis

Data will be analyzed via SPSS. We will conduct ANOVAs to examine between group (meditation vs. control) differences post-intervention controlling for baseline values for our primary, secondary, and tertiary outcomes. We will covary for relevant variables, including age, sex, and education level, which are known to impact measures of cognitive function. We will use the intent-to-treat model, where all participants will be included in our statistical analyses regardless of whether they completed the intervention or not.

## Discussion

Our proof-of-concept study has the potential to unveil an affordable, feasible, and scalable intervention to improve attention and mobility in older adults at-risk for falls. While the relationship between attention and falls risk has been examined in numerous cross-sectional studies, research aiming to directly improve attention through an intervention are limited. We acknowledge the potential barriers towards the success of our study which are discussed below.

First, because this study is the first to examine whether meditation training will improve attention in older adults, it is possible that our intervention will not be long enough to observe changes in attention. Because meditation interventions are relatively new in the literature, there is no standardized protocol that has been developed; additionally, research examining the dose-response relationship between meditation and cognitive benefits is lacking. Therefore, our 4-week intervention was chosen based on previous studies that have found medium to large effect sizes for studies between one single session [16] and 2 months [19, 33]. Future studies examining the dose-response relationship between meditation and attention in this particular population are warranted.

Second, we acknowledge that our intervention designed to improve attention in older adults might not translate to changes in mobility and/or a reduction in future falls. However, this will be among the first studies to examine the potential causal role of attention in falls risk—in contrast to the correlational studies that currently dominate the literature. While our current study is not specifically aimed at improving mobility or reducing falls per se, our study provides the groundwork for future definitive RCTs aimed at examining the efficacy of meditation for reducing falls risk in older adults.

## Conclusion

Meditation has been shown to provide a host of benefits for a variety of populations. Our research aims to examine whether meditation can improve attention—a hallmark indicator of falls risk—in our aging population.
